# Overview of a formal scoping review on health system report cards

**DOI:** 10.1186/1748-5908-5-2

**Published:** 2010-01-15

**Authors:** Susan E Brien, Diane L Lorenzetti, Steven Lewis, James Kennedy, William A Ghali

**Affiliations:** 1 Department of Community Health Sciences, Faculty of Medicine, University of Calgary, Calgary, Canada; 2 Institute of Health Economics, Jasper Avenue, Edmonton, Canada; 3 Access Consulting Ltd, Saskatoon, Canada; 4 Experimental Medicine Division, John Radcliffe Hospital, University of Oxford, Oxford, UK

## Abstract

**Background:**

There is an extensive body of literature on health system quality reporting that has yet to be characterized. Scoping is a novel methodology for systematically assessing the breadth of a body of literature in a particular research area. Our objectives were to showcase the scoping review methodology in the review of health system quality reporting, and to report on the extent of the literature in this area.

**Methods:**

A scoping review was performed based on the York methodology outlined by Arksey and O'Malley from the University of York, United Kingdom. We searched 14 peer reviewed and grey literature databases limiting the search to English language and non-English language articles with English abstracts published between 1980 and June 2006 with an update to November 2008. We also searched specific websites, reference lists, and key journals for relevant material and solicited input from key stakeholders. Inclusion/exclusion criteria were applied to select relevant material and qualitative information was charted from the selected literature.

**Results:**

A total of 10,102 articles were identified from searching the literature databases, 821 were deemed relevant to our scoping review. An additional 401 were identified from updates, website searching, references lists, key journals, and stakeholder suggestions for a total of 1,222 included articles. These were categorized and catalogued according to the inclusion criteria, and further subcategories were identified through the charting process. Topic areas represented by this review included the effectiveness of health system report cards (n = 194 articles), methodological issues in their development (n = 815 articles), stakeholder views on report cards (n = 144 articles), and ethical considerations around their development (n = 69 articles).

**Conclusions:**

The scoping review methodology has permitted us to characterize and catalogue the extensive body of literature pertaining to health system report cards. The resulting literature repository that our review has created can be of use to researchers and health system stakeholders interested in the topic of health system quality measurement and reporting.

## Background

Health system quality reporting refers to measuring healthcare service provision (*i.e*., care provided in hospitals, clinics, the community, and public health) and comparing these measurements to benchmarks or other standards to determine if best practices are being used and/or resources are being used efficiently. The results, in the form of report cards, are fed back to health service providers and sometimes other groups to potentially change practice patterns to improve effectiveness and efficiency of care. Reporting on health system quality has become a common tool to increase accountability, improve efficiency, determine funding, and attract consumers in many healthcare systems worldwide [1-4]. As a result, there has been a substantial increase in the body of literature regarding these report cards. However, the extent of the literature and specific topics described are unclear, and evidence-based standardized methodologies for the creation of health system quality report cards have yet to be established. Indeed, healthcare and public health policy makers, managers, and administrators have few consensus documents or evidence-based examples of effective and accepted means of health system quality reporting programs.

Due to the importance of health system quality reporting as a mechanism for accounting to patients, the public, governments, and funding sources, it is imperative that report cards be valid and accurately reflect the quality of healthcare being provided. Standardized practices for collecting and analyzing data must be developed, along with appropriate methods for reporting results to different stakeholders. The effectiveness of report cards in changing practice needs to be evaluated, and potential improvements must be identified to ensure that they improve care. Also, gaps in current research knowledge need to be identified to guide future research. Thus, clarification and understanding of existing literature on health system quality report cards is the first step in addressing these concerns. Adequately disseminating the research findings to health services researchers, healthcare providers, and administrators will promote evidence-based reporting on the quality of healthcare services in the future.

Scoping reviews are a relatively new type of research review that provide a tool for summarizing literature in a topic area such as health system quality reporting [5]. Scoping reviews are somewhat similar to systematic reviews in that they are used to methodically organize and describe a body of literature. However, there are several aspects of scoping reviews that distinguish them from traditional systematic reviews (Table 1). Systematic reviews attempt to answer a clearly defined question, and often use explicit methodologies to asses the quality of included articles. In contrast, scoping reviews are generally conducted to examine the extent, range, and nature of research activity in a particular field, without necessarily delving into the literature in-depth or attempting to assess its quality. Scoping reviews produce a profile of the existing literature in a topic area, creating a rich database of literature that can serve as a foundation for more detailed reviews. These reviews are not intended to assess the quality of the existing literature, but may provide the background for full systematic reviews in a research area, or identify areas in the literature where existing research is sparse.

**Table 1:** A comparison of the characteristics of scoping and systematic reviews.

Systematic Review	Scoping Review
Focused research question with narrow parameters	Research question(s) often broad

Inclusion/exclusion usually defined at outset	Inclusion/exclusion can be developed *post hoc*

Quality filters often applied	Quality not an initial priority

Detailed data extraction	May or may not involve data extraction

Quantitative synthesis often performed	Synthesis more qualitative, and typically not quantitative

Formally assesses the quality of studies and generates a conclusion relating to the focused research question	Used to identify parameters and gaps in a body of literature

The comprehensive nature of scoping reviews provides a mechanism to thoroughly and systematically map the existing literature regarding health system quality report cards. Here, we summarize the findings of a scoping review conducted in response to a Canadian Institutes of Health Research (CIHR) call for special policy-relevant research syntheses and overviews. The CIHR's call for scoping review studies identified a number of priority topic areas, including health system performance evaluation, which had previously been identified by national multidisciplinary and multi-sectoral health system stakeholders to be areas of special interest. Our scoping review's aim was to document and catalogue the extent of published material relating to the production, reporting, and dissemination of health system report cards. This paper demonstrates the steps taken to conduct our scoping review, and outlines a conceptual categorization of the large body of literature on healthcare quality reporting.

## Methods

Arksey and O'Malley from the Centre for Reviews and Dissemination at the University of York published a pivotal paper in 2005 on the conduct of scoping reviews [5] that provides a methodological framework to carry out this type of review. This 'York framework' suggests five stages that we followed for this review: Identification of the research question to be addressed; identification of studies relevant to the research question; selection of studies to include in the review; charting of information and data within the included studies; and collating, summarizing and reporting results of the review. An optional sixth stage involves consultation with stakeholders to ensure comprehensive inclusion of all relevant material [5]. We used this template to guide our scoping review, and where necessary, developed more specific procedures to carry out the stages of the review process. The ensuing sections describe the methods we followed in our scoping review of the health system report card literature.

### Development of research question

The York framework recommends that in the development of the research question, all aspects of the research area should be considered to generate a breadth of coverage. Drawing on the expertise of our research team and an initial scan of the literature, we defined our overriding research question as follows: What is the extent of published evidence on best practices relating to the production, reporting, and dissemination of health system report cards? The rationale behind this broad question was the increased use of health system quality reports and the apparent lack of consensus in the literature on how best to design them. Although extensive, the existing literature on health system report cards is heterogeneous in its areas of focus and also its methodological rigor, with, for example, an abundance of quasi-experimental evaluations and studies [6]. Therefore, based on a combination of informal discussions, preliminary review of published topics and stakeholder consultation (see below), we developed the following focus areas for our scoping review:

1. Methodological issues in health system report card development. Specific examples of methodological issues that have been addressed at least to some extent in the literature include: What data sources can be used for studying quality of care? Does the accuracy of process and outcomes of care measurements vary across different data sources? What is the best approach to developing and validating quality indicators in specific clinical areas? What clinical areas have published widely endorsed and/or applied quality indicators? What statistical methods should be used to risk-adjust data in health outcome report cards? What is the optimal format for presenting and reporting outcome or process data? Do data framing effects influence reactions to data presented in the reports? How, and to whom, should health system reports be disseminated? What are the pros and cons of public reporting relative to reporting to providers only, or providers and health system administrators?

2. Evidence of effectiveness/efficacy of report cards for enhancing quality. More specifically, do report cards actually affect quality of care and outcomes?

3. Research into stakeholder views of report cards. What opinions do the general public, providers, and health system decision makers have of health system report cards? Do health system report cards influence the decision making of the various players (*i.e*., patients, providers, and/or decision makers) in the health system?

4. Ethical considerations relating to report cards. How should providers respond to demands for accountability? What are the ethical considerations regarding public reporting of quality of care outcomes? Do health system report cards have any detrimental effects on access to care for marginalized groups?

### Stakeholder consultation

The optional stakeholder consultation phase is meant to be an ongoing interaction throughout the review process [5]. Thus, we felt it was important to initiate contact with stakeholders at the beginning of the review process. Early involvement of stakeholders allowed us to seek guidance regarding our research question and choice of focus areas, thus ensuring that the results are of broad interest among different stakeholder groups.

We identified fifteen stakeholders representing fellow researchers, decision makers, and clinicians involved in health system quality reporting. We contacted these individuals via email, and briefed them on our research question and focus areas and approach to searching the literature, and solicited their feedback on our approach. Ten of the fifteen stakeholders expressed interest in the study and provided us with valuable input. They deemed our research question and focus areas to be suitable and broad enough to address the research question, and suggested appropriate studies to include. The stakeholders confirmed the need for this review, and provided suggestions as to how best distribute our knowledge and research products to various stakeholder groups (see the Discussion section for more information on stakeholder engagement in the dissemination phase of our work).

### Search strategy

To be comprehensive, the York framework recommends searching several literature sources, including electronic databases, reference lists of relevant literature, hand-searching key journals, and existing networks, relevant organizations, and conferences [5]. For our scoping review, we approached this in multiple steps, first targeting electronic literature databases. Once relevant material was selected from this source, we then searched relevant websites, URLs, and reference lists of key studies to increase our capture of relevant material.

### Electronic literature database searching

We enlisted the services of a library scientist (DLL) to conduct the electronic database search. The research team devised a broad list of terms pertinent to health system report card research, including report cards, performance indicators, scorecards, system performance, quality improvement, health, healthcare, and medical care. These terms were combined to create keywords that could be used to search both peer-reviewed and grey literature electronic databases: quality indicators, healthcare AND reports/reporting, quality of healthcare AND reports/reporting, benchmarking AND reports/reporting, report card/cards AND health/healthcare/medical, performance reports/reporting AND health/healthcare/medical care, quality reports/reporting AND health/healthcare/medical care, health system evaluation/quality/performance/rating, health system reports/reporting, healthcare evaluation/quality/performance/rating AND reports/reporting, healthcare system reports/reporting, consumer reports AND health/healthcare/medical care, public performance reports/reporting AND health/healthcare/medical care, public reporting AND health/healthcare/medical care. Keywords were then mapped to database thesauri search terms, where available, and were also searched as text word terms in all databases. The goal was to conduct a sensitive rather than specific search of the literature; thus search terms were of necessity kept very broad, resulting in many irrelevant studies being eliminated at the study selection phase (see below).

A total of 14 peer-reviewed and grey literature databases were searched using these search strings. The peer-reviewed databases searched were: ABI Inform, Cumulative Index of Nursing and Allied Health Literature (CINAHL), Cochrane Library, EconLit, EMBASE, MEDLINE, PsycINFO, and Social Sciences Abstracts. The grey literature databases searched were: Grey Literature Report. http://www.nyam.org/library/greyreport.shtml, PapersFirst, ProQuest Dissertations and Theses, University of York Health Technology Assessment (HTA) database http://www.crd.york.ac.uk/crdweb/, University of Laval KUUC Knowledge Utilization Database http://kuuc.chair.ulaval.ca/english/index.php, and WorldCat.

All literature database searches were limited to the English language and non-English language articles with English abstracts, and published between 1980 and June 2006. The literature search was subsequently updated to November 2008.

### Website searching

Once the relevant studies were selected from the literature database search, we carried out a selective search of relevant websites. Through consultation with our stakeholders, and members of the research team and colleagues, we compiled a list of relevant websites to search (Table 2). We attempted to search websites in a systematic manner, allowing for some variation in search strategies in response to varied website structures. For example, most websites provide research and/or publication links which contain a central repository of an organizations reports, research papers, and/or publications. However, other websites have this material scattered throughout, making it more difficult to uncover. Therefore, our first approach to a website was to consult the site map and look for research and/or publication links. For websites without this link, we took a more sporadic approach, checking all the links for relevant material.

**Table 2:** List of Organizations included in the targeted website searching

Europe	United States
European Centre for Health Policy	Center for Studying Health System Change

European Centre for Social Welfare and Policy Research	Centers for Medicare & Medicaid Services

Health Impact Assessment Database	Health Policy Institute

International Health Policy Library	National Center for Policy Analysis

International Network of Agencies for Health Technology Assessment	RAND Organization

World Health Organization	U.S. Department of Health and Human Services

**Australia**	U.S. Agency for Healthcare Research and Quality

Australia Health and Aging	U.S. Dept HHS National Institutes of Health

Australian Policy Online	U.S. Dept. Veteran Affairs

Centre for Clinical Effectiveness (Monash University)	**Canada**

Centre for Health Economics (Monash University)	Centre for Health Services and Policy Research

Monash Institute of Health Services Research	The Fraser Institute

**United Kingdom**	Institute for Clinical Evaluative Sciences

Centre for Health Economics (University of York)	Manitoba Centre for Health Policy

Centre for Reviews and Dissemination, University of York	

Institute for Public Policy Research	

King's Fund	

National Institute for Clinical Excellence	

Policy Studies Institute	

UK National Health Service	

UK Health and Wellbeing	

UK National Research Register	

Once hand searching a website's links was complete, we used the website's search engine to attempt to uncover additional material. Once again, different types of search engines required different search tactics. For all websites, we searched the terms 'healthcare quality,' 'performance report,' and 'report card'. For websites that were not specifically healthcare-focused, we added the word 'health' to the specific term. We kept a log of the website searches, saving the links to relevant pages and tracking our progress through the websites.

### Other literature sources

In an attempt to be as comprehensive as possible in our search, we also collected literature from reference lists of relevant articles, specific journal issues with related material, and suggestions from colleagues.

### Study selection

Our employ of broad terms in the electronic database searches generated a list of over 10,000 abstracts. In order to sort out the irrelevant material from this list, we developed a screening tool with specific inclusion and exclusion criteria based on the focus areas identified with our research question. Three members of the research team piloted the inclusion/exclusion criteria with a sub-sample of abstracts retrieved from the MEDLINE database. Multiple sample tests of criteria were carried out, and feedback from these tests was used to refine the abstract screening process. Once a final set of inclusion/exclusion criteria were agreed upon, the inter-rater reliability for this process was confirmed using a kappa analysis of 35 abstracts (kappa = 0.79).

One member of the research team was responsible for reading the abstracts of all the articles identified in the search of electronic database, applying the inclusion/exclusion criteria in the abstract screening tool. For inclusion in the scoping review, the abstracts had to indicate that the articles contain: Original research (including systematic reviews) on 1) efficacy or effectiveness of health system report cards or 2) stakeholder views of health system report cards; or original research and/or a focused discussion of 3) ethical considerations or 4) methodological approaches to health system report cards. In addition to peer-reviewed articles, we also included research reports, theses, and policy analyses if they met the other inclusion criteria.

Excluded from the review were obvious commentaries, editorials, or non-systematic reviews regarding health system report cards (except for inclusion criteria three and four), articles describing the audit of a particular healthcare service, but lacking the feedback component of the report card process, and articles on non-healthcare-related quality reporting.

A similar screening process was used for literature uncovered through website searching, reference lists, and recommendations. For material from websites, less formal, interpretive descriptions of a study or investigation that may be on a home page or a web page that may or may not be linked to the report document were also excluded.

### Charting

According to the York methodology of scoping reviews, the charting process is multi-staged, involving extraction of information from individual articles. We collected descriptive characteristics such as general citation information, clinical area, level of reporting, country of origin, and key findings from the included articles to create a detailed spreadsheet database.

### Summation, collation, and synthesis

The purpose of this final stage of scoping is to provide a structure to the literature uncovered. Due to the broad scope of our research question and the subsequent large volume of literature uncovered in our searches, we contained this final stage to a narrative synthesis where we organized these findings into specific categories based on our focus areas and abstract screening tool: evidence of effectiveness of report cards; stakeholder views of report cards; methods associated with report cards; ethical considerations for report cards. Focusing on the descriptive nature of the material in the charting phase allowed for the identification of additional categories and themes in the literature. Creation of these *a priori *sub-categories provided a structure to the findings and a clearer way of describing the literature.

## Results

### Overview of results

**Figure 1:**
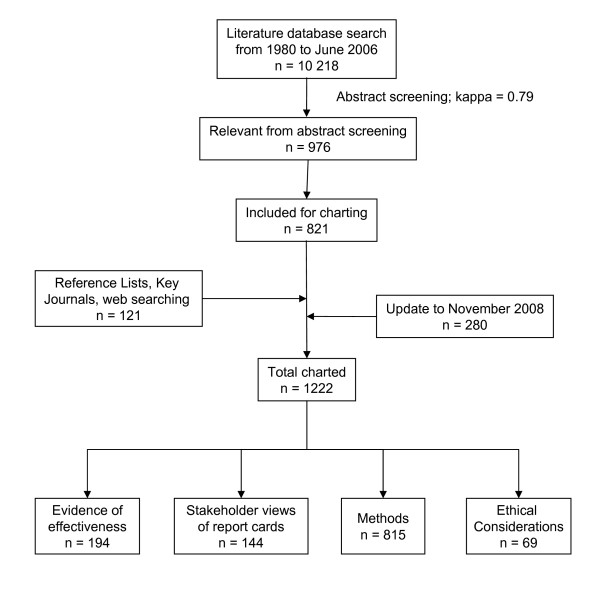
**Flow diagram of the progression of information through the scoping review into categories**.

A total of 10,218 articles were initially identified as potentially relevant from our search of the peer-reviewed and grey literature electronic databases. Using the abstract screening tool, 976 articles were retrieved for charting. Of these articles, 821 were read in more detail and charted. An additional 121 items from website searching and other sources (*e.g*. reference lists) were charted for a total of 942 articles in the initial round of searching. Updating the electronic databases search yielded an additional 3,014 articles, of which 280 were charted, for a total of 1,221 articles charted. Each of the selected articles were categorized into the four focus areas: evidence of report card effectiveness in improving the quality of healthcare (n = 194); stakeholders' opinions, views and understanding of report cards (n = 144); articles addressing various methods (*e.g*., statistics, data sources, quality indicators, data display, distribution) of report cards (n = 815); and ethical considerations or issues that have arisen due to health system report card use (n = 69; Figure 1).

### Countries of origin

The majority (65%) of material uncovered originated from the United States (US), where there is a culture of healthcare report cards. Approximately 11% of the material originated from the United Kingdom (UK), 4% from Europe, 2% from Scandinavia, and 7% from Canada. Another 2% originated from Australia and New Zealand, and 1.3% from Asia. Approximately 5% came from miscellaneous countries such as Israel, United Arab Emirates, Brazil, Mexico and several African countries. Publications from the World Health Organization and the Organisation for Economic Co-Operation and Development each accounted for about 1% of the literature found.

### Level of healthcare quality reporting

The majority of the literature pertained to four broad levels within healthcare: system, facility, group, or individual. More specifically, 328 articles pertained to quality reporting at the level of healthcare system or healthcare plan. Four hundred and forty-three articles described an aspect of quality reporting at the level of hospitals or other healthcare facilities (*e.g*., nursing home, long-term care facility or psychiatric facility). One hundred and fifty-nine articles focused on quality of care provided by groups of healthcare providers such as clinical departments, groups of physicians, nurses or other providers (*e.g*., therapists), and 167 articles pertained to healthcare quality provided by individual physicians, nurses, or other providers. Twenty-two articles spanned two or more of these healthcare levels, and for 125 articles it was either not an applicable or relevant categorization (*e.g*., a specific statistical model) or it was not immediately apparent at what level the article reported on healthcare quality.

### Clinical areas represented

It was noted that particular clinical areas had larger volumes of literature pertaining to performance reporting. Table 3 describes the most common clinical areas found in the literature. The category of cardiac care/cardiac surgery includes articles describing healthcare quality provided to treat conditions such as acute myocardial infarction, heart failure, or outcomes following cardiac interventions (*e.g*., angioplasty) or cardiac surgery (*e.g*., bypass surgery). 'Mental healthcare' includes in-patient and out-patient psychiatric care. 'Surgery' includes articles pertaining to quality of surgeries other than cardiac or oncologic surgeries. 'Oncology/cancer care' includes articles describing outcomes following surgical oncology procedures and screening for different types of cancer (*e.g*., cervical, colon).

**Table 3:** List of the most common clinical areas in healthcare quality reporting literature

Theme	Number of articles
Cardiac care/cardiac surgery	127

Primary care/general practice	59

Mental healthcare	48

Nursing home care/long term care	42

Surgery	41

Obstetrical care	23

Geriatrics	22

Diabetes care	21

Pediatric/neonatal	20

Public health	18

Oncology/cancer care	17

Not all literature included in the review pertained to a specific clinical area. Furthermore, some literature pertained to more than one clinical area, such as nursing home care and geriatrics.

### Common groups and projects

Several organizations commonly reported research pertaining to their healthcare performance measurement and quality improvement initiatives. As expected, many of these organizations are based in the US, several of which are agencies within the federal government departments. For example, within the US, the Department of Health and Human Services, the Agency for Healthcare Research and Quality, the Centers for Medicare and Medicaid Services and Prevention, and the Centers for Disease Control all have healthcare performance reporting and quality improvement initiatives that were uncovered in the scoping review. Within the US Department of Veterans Affairs, the Veterans Health Administration is also involved in numerous quality-of-care monitoring and performance improvement initiatives.

There are also several non-governmental organizations in the US that published material pertaining to our scoping review focus areas, including the Joint Commission on Accreditation in Healthcare, the National Committee for Quality Assurance and RAND Health. Specific projects based in the US, separate from these and federal government organizations and prevalent in the selected literature include the New York State Cardiac Surgery Reports, the Northern New England Cardiovascular Disease Study Group, the Pennsylvania Consumer Guide to Coronary Artery Bypass Graft Surgery, the Cleveland Health Quality Choice Coalition, the Nursing Outcomes Coalition, and the National Database of Nursing Quality Indicators.

Outside of the US, a few other groups and projects were reported several times in our selected literature. From the UK, the Healthcare Commission published material pertaining to methods and evidence of effectiveness of healthcare report cards utilizing data from the National Health Services (NHS). The Australian Council on Healthcare Standards reported on the development of comparative indicators. The Institute of Clinical Evaluative Sciences based in Canada has also published several reports pertaining to report card methods. On an international level, the World Health Organization published material pertaining to report card framework and statistical analyses and the Organization for Economic Cooperation and Development published material pertaining to quality indicator development.

### Findings within focus areas

Below we provide an illustrative overview of the information identified in each focus area. For each topic area, we do not cite all identified references because of their large number. Instead, we refer readers to the corresponding topic tabs in the literature database for our scoping review (Additional File 1).

### Evidence of effectiveness

We identified a total of 194 articles addressing the question of effectiveness of health system report cards. These are listed in the online literature database behind the 'evidence of effectiveness' tab. These articles include many that assess the influence of report cards on patient or purchaser choices relating to healthcare providers and services. This general issue is also assessed to some extent in the articles identified in the stakeholder views topic area (discussed below).

A number of other identified articles assess the impact of health system reports on quality of care, with quality measured using a variety of indicators including outcome and process measures. This body of literature includes a Cochrane Collaboration systematic review by Jamtvedt *et al*. [7] focusing on the effect of audit and feedback on health system performance.

### Stakeholder views

We identified a total of 144 articles reporting on the views held by various stakeholders regarding health system report cards. This includes studies focusing on consumers (*e.g*., patients and purchasers), physicians, other healthcare providers (*e.g*., dentists, nurses, therapists), and healthcare managers. There were generally three areas of focus in the studies that we identified: stakeholder opinions of report cards; their understanding of report card information; and how they use the information in report cards to make decisions.

### Ethical considerations

Studies on ethical considerations of report cards were not abundant, with 69 articles identified. The most commonly discussed topics were the unintended consequences of report cards, and more specifically, the impact of report cards on vulnerable patient populations. Discussions of ethical considerations of pay-for-performance schemes or performance-based contracting (*i.e*., where provider payment is linked to performance) were also uncovered in our literature search update. The clinical area of cardiac care (*i.e*., cardiac surgery, procedures, cardiology care) has undergone considerable report card activity, and thus the majority of articles within this focus area are in the context of cardiac care. In particular, the ethical impacts of the highly-publicized surgeon-specific New York State Coronary Artery Bypass Graft Report are most commonly discussed. There are also several publications that discuss ethical frameworks for report cards. Once again, cardiac care report cards were most often represented. However an ethical framework for mental health care quality reporting was also published.

### Methodology

**Figure 2:**
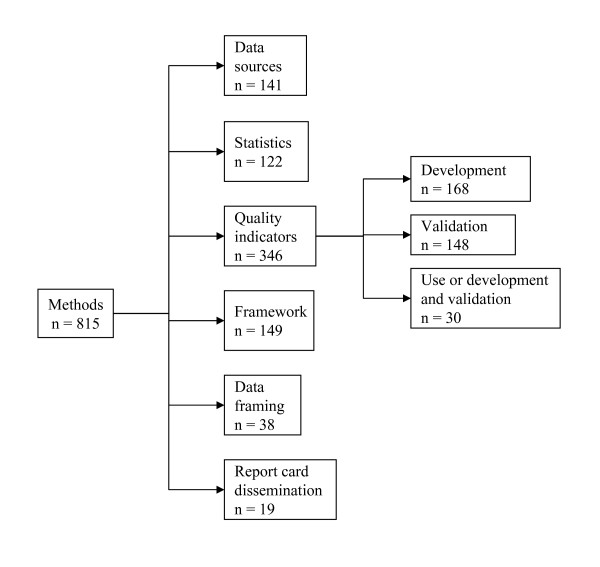
**Categorization of material pertaining to health system report card methodologies**.

We identified a total of 815 articles focusing on methodologies for health system report cards. This largest group of articles and studies was further divided into the following categories (Figure 2): articles examining dissemination of report cards (n = 19), those discussing how data are presented or framed (n = 38), descriptions of different frameworks for report cards (n = 149), data sources for report cards (n = 141), statistical methods used to create report cards (n = 122), and the measures or quality indicators used in report cards (n = 346).

### Data framing and report card dissemination

The framing, or display of data, and the manner in which a report card is distributed are both important aspects of the report card process, yet the literature regarding these aspects was sparse. With respect to data framing, most of the material discussed consumer comprehension of report card data. Several articles presented different methods of report card display for a variety of audiences: spider diagrams and dashboards for reporting data to administrators in an understandable format, statistical process control, and league tables.

Material published regarding the dissemination of report cards focused on public reporting. Some articles discussed the impact and outcome of public versus private reporting. Others discussed the development and design, use, and comprehension of web-based public reporting.

### Report card framework

There are several formal frameworks around which some, but not all, health service quality report cards are based. The scoping review uncovered publications discussing the use of the balanced scorecard approach, the Donabedian model, statistical control charts, and the Baldridge Quality Criteria in development of report cards. These as well as other less formal models for report card programs were presented for measuring quality of care in healthcare systems, hospitals, and health plans. Furthermore, models for performance reporting in the clinical areas of cardiovascular health, mental health, primary care, and long-term care were presented. The updated literature search also identified frameworks for pay-for-performance programs.

### Data sources

A total of 141 identified articles address methodological considerations surrounding the data sources used to produce health system report cards. Report cards can be based on a variety of data sources, including administrative sources, prospective clinical data collection, retrospective chart abstraction, patient survey or interviews, and/or provider interviews and reports. Several prospective clinical data collection systems for the purpose of monitoring performance for quality improvement were described along with patient survey methods, and administrative data.

However, the majority of the literature compared or validated data sources, and the most common comparisons were made between administrative data and clinical data. Patient surveys also were validated and compared against administrative data and provider reports. Some unique data sources also were compared to more standard sources; the use of clinical vignettes to measure performance was validated against chart abstraction and standardized patients in one study. Finally, some studies compared the performance of quality measurement systems that use only a single data source relative to systems that use a combination of data sources to create more complete databases.

### Statistical methods

Our review identified 122 studies addressing statistical methods for health system report cards. These primarily focused on risk adjustment methods. Earlier material (*i.e*., from the 1990s) discussed and argued for risk adjusting rather that the use of raw rates in order to produce performance measures that accurately reflected quality of care and could be used for comparisons. However, some more recent articles addressing the same issue were also found suggesting that despite earlier work demonstrating the need for risk adjustment, some performance measurements and report cards still do not risk adjust sufficiently.

Methods for risk adjustment discussed in the literature include hierarchical models, fixed effects, random effects and standard logistic regression models, P-charts, receiver operating characteristics curve analysis, and standard deviation calculation methods. Risk adjustment using generic severity indices, such as the Charlson Comorbidity Index, or the Acute Physiology and Chronic Health Evaluation (APACHE) score, were discussed in some of the identified articles. Risk adjustment for patient characteristics, and specifically socio-economic factors, were addressed, as were risk adjustments for hospital characteristics, such as peer group, acceptance of patient transfers, number of emergency surgeries, or the institutional protocols regarding do not resuscitate orders. Adjustment methods for specific data types, such as administrative data or patient surveys, were also addressed.

### Quality indicators

This subsection of the Methods focus area identified a total of 346 studies. This group of studies was further subdivided into three groups: quality indicator development (n = 168), quality indicator validation (n = 148), and uptake or combined development and validation of quality indicators (n = 30).

### Quality indicator development

Publications in this topic area described the development of quality indicators in a variety of healthcare settings and clinical areas. Quality indicator development for overall hospital performance, health system performance assessment, and consumer/patient satisfaction with care were commonly covered in the literature. There was substantial literature regarding the development of performance indicators for cardiac care, surgery outcomes, mental health, and nursing homes/long-term care. Some indicators for cancer care were also found. Other clinical areas with quality indicator development include nursing care, mental health treatment, surgery, primary care, and public health.

Many of the indicator projects described above utilized the Delphi method or a modification of the technique for quality indicator development. However, other methods have been used to develop quality indicators, including modified nominal group technique, the RAND Appropriateness Method (*i.e*., a combination of Delphi panel and nominal group technique), and adaptation of indicators from clinical guidelines.

### Quality indicator validation and uptake

Literature catalogued in this subcategory was generally focused on the validity of outcomes measures as quality indicators. Studies focusing on the validation of mortality rates, readmission rates, and patient satisfaction surveys were also found. Several articles debating the use of structure, process, and outcomes measures were uncovered, and the earliest of these recommended the use of the Donabedian approach in measuring quality of care. A subset of studies identified also assessed the extent to which validated quality indicators developed and applied in one country or jurisdiction could be used in other settings or countries.

## Discussion

Using the methodology described in the York Framework and methods developed specifically for our review, we uncovered a large volume of peer-reviewed and grey literature pertaining to the published evidence pertaining to the production, reporting, and dissemination of health system report cards. We have outlined a framework for the existing literature, and through the charting process we have created a comprehensive, catalogued database of the literature (Additional File 1) that is useful for future research on health system quality reporting. We also contribute to the methodological literature of scoping reviews by describing in detail our review protocol and our specific approach to a targeted search of the internet for relevant material.

We found numerous articles pertaining to the methodology for producing health system report cards; in particular, we catalogued an extensive database on the development and validation of quality indicators. We also uncovered a considerable volume of literature on data sources used to produce report cards, and several statistical models for risk adjusting outcome performance indicators. The majority of health system report card literature originated from the US, and the report card activity of several groups were repeatedly represented in the literature.

Similar volumes of literature were uncovered for quality reporting at the system, hospital, and provider levels, indicating that the practice of quality reporting is occurring throughout the different levels of healthcare. Finally, our results reveal that certain clinical areas, such as cardiac care, cardiac surgery, and primary care or general practice, have greater report card activity relative to other clinical areas (*e.g*., cancer care). For the clinical areas still in the preliminary stages of developing quality report cards, it is hoped that report card developers look to clinical areas with more advanced activity to draw on their experiences and avoid 'reinventing the wheel' of report card development.

### Challenges and limitations

This was our first encounter as a research team with the scoping review methodology, and it is important to discuss our experience of using the methodology. Scoping is a relatively new review method and that has been embraced by several research and granting organizations as a rapid method for mapping and synthesizing existing literature in a particular topic area and identifying gaps where future research should be conducted. However, we experienced several shortcomings with the methodology, and challenge several of its purposes as described by Arksey and O'Malley.

First, although we created a comprehensive database of existing literature on health system quality report cards, this was by no means a rapid process (taking more than a year to complete). The volume of literature that we amassed in this scoping review is so great (*i.e*., over 1,200 relevant articles uncovered) that it is not feasible to chart articles in more depth, while still maintaining the breadth of perspective required for scoping. Others have published scoping reviews with smaller volumes of relevant literature that contain succinct, detailed syntheses of the uncovered literature [7-9]. Such comprehensive synthesis of the literature was not practical for the volume of literature that we uncovered in our scoping review. Thus, recognizing the breadth of our literature scan, we opted to produce a catalogued database of the literature that can be accessed electronically to perform more in-depth research on specific topic areas.

Secondly, by definition, scoping reviews are not intended to assess the quality of the literature scoped. Therefore, it is difficult to identify where the literature is lacking regarding a given research topic without assessing the quality of the existing literature. The existence of published material in a particular topic area does not necessarily provide sufficient evidence to base decisions [10]. Thus, in the case of scoping reviews that uncover a volumes of material too large for further syntheses to be practical, this review type is best suited to identify volumes of literature and categorize the material by common themes and topics, thus helping to identify where further syntheses can be efficiently carried out. This lack of quality assessment of the literature is difficult to reconcile and can create difficulties with the understanding and acceptance of this review type. Indeed, scoping reviews are often misinterpreted to be a less rigorous systematic review, when in actual fact they are a different entity.

In addition to the methodological issues we experienced with scoping reviews, several operational limitations also arose. Scoping reviews provide information on the scope of a body of literature at only a single moment in time. Hence, they are, in essence, out of date shortly after their completion. As we experienced, the task of updating comprehensive scoping reviews is not small, and can not be readily undertaken by research groups without the perpetual availability of ongoing resources and personnel. In this regard, web 2.0 'wiki' auto-updating is a mechanism that could be explored in future research surrounding scoping reviews.

In addition, on some levels, we are uncertain about the utility of large-scale scoping reviews to stakeholders. The packaging of a large volume of literature into a catalogued database may be useful to researchers; however, it is unclear whether policy makers or administrators would use such a resource. Greater synthesis of the results would create a more distilled product more suitable to policy and decision maker use, although it is difficult to see how a huge body of literature can be distilled into any representative product. Despite these caveats, we have provided narrative text in the results section above that presents representative information on what our scoping review has yielded. We have also made the catalogued literature database available to all interested parties, and can now proceed to conduct targeted systematic reviews and meta-analyses to distill the information into more useable formats.

Another general challenge is that of knowledge translation. We have undertaken stakeholder-targeted dissemination activities of our findings that may contribute to collective knowledge of various aspects of health system report cards. Since completion of our review, we have interacted with various stakeholders (*i.e*., a combination of health services researchers, health system analysts involved in quality of care reporting, and health system decision makers) at information sharing events relating to the Canadian Cardiovascular Outcomes Research Team (CCORT--see http://www.ccort.ca), the Society for General Internal Medicine http://www.sgim.org, and the National meeting of the national APPROACH network (see http://www.approach.org). Further engagement of stakeholders has also occurred through the sharing of a scoping review report with the Institute for Health Services and Policy Research at the Canadian Institutes of Health Research. Knowledge exchange relating to our work will also be enhanced by the academic dissemination of our findings through this article and its accompanying on-line literature database.

Finally, as scoping reviews become more common, there is the challenge of determining the quality of the review. Guidelines exist for the reporting of many types of health research studies. Such guidelines have been developed to help ensure quality and transparency of health research http://www.equator-network.org/home/. However, guidelines for scoping reviews do not exist currently. Herein we have presented a particular format for reporting a scoping review. We loosely followed the existing template for systematic reviews (Transparent Reporting of Systematic Reviews and Meta-Analyses, PRISMA; http://www.prisma-statement.org/index.htm). Development of guidelines to aid in the reporting of scoping reviews would improve their transparency and perhaps acceptance in the medical literature.

## Summary

Scoping reviews may be useful for mapping literature and identifying where more in-depth reviews and syntheses can be carried out. The timeliness and depth of the scoping review results is dependent on the volume of literature that exists in the particular topic area to be scoped. Our scoping review has produced a comprehensive literature database from a large body of literature pertaining to health system quality report cards. The resulting literature repository that our review has created may be of use to researchers and health system stakeholders interested in the topic of health system quality measurement and reporting.

## Competing interests

The authors declare that they have no competing interests.

## Authors' contributions

SB participated in the design and coordination of the study, carried out the website searching, title and abstract review, article review, collation and organization of material, statistical analysis, drafted and revised the manuscript. DL participated in the design and coordination of the study, carried out the database literature searches, website searching, title and abstract review and helped to draft the manuscript. SL participated in the design and coordination of the study. JK helped with the conception of the study and participated in the design and coordination of the study. WG conceived of the study, secured funding for the study, participated in the design and coordination of the study, participated in some title abstract review, and helped draft and revise the manuscript. All authors read and approved the final manuscript.

## Supplementary Material

Additional file 1 **Reference Database**. This file contains the references selected from the scoping review of the literature pertaining to health system report cards. The file contains tabs that are labeled according to the categories listed in Figures 1 and 2, and the references are organized under these categories. We list an identification number, first author and year of publication, title, citation, country of origin, level within health care system at which the reporting occurs (e.g. system level, hospital, individual provider, etc.), clinical area, category and sub categories. Click here for file
